# Supplementary material for: Tumor diversity and the trade-off between universal cancer tasks
Source: Nat Commun. 2019 Nov 28;10:5423. doi: 10.1038/s41467-019-13195-1 (PMC6882839; doi:10.1038/s41467-019-13195-1)
Supplement: Supplementary file 1 — Supplementary Information [file 41467_2019_13195_MOESM1_ESM.pdf]

Supplementary Information

Tumor diversity and the trade-off between universal cancer tasks.

Hausser et al.

Supplementary Notes

Supplementary Note 1 - The observation that tumors are not found in the immediate vicinity of archetypes is an artifact of visualizing high-dimensional data

In examining polyhedra of dimension three or higher (4 archetypes or more), we can observe that there are no tumors in the direct vicinity of the archetypes. This observation has a statistical explanation: measurement error tends to push archetypes away from the data. In addition, projecting higher dimensional polyhedra onto a plane – a step necessary for visualization – artificially increases the density of generalists compared to specialists: the more archetypes, the higher the dimension, and the smaller the density of projected data close to the archetypes.

To illustrate this, we simulate data points by sampling uniformly from polyhedra with 3, 4, 5 and 6 archetypes (Supplementary Fig. 2E). After adding measurement noise, determine the position of archetypes using ParTI and project the data onto the face of the polyhedron defined by the first 3 archetypes.

As the panel with 3 archetypes shows, adding noise pushes the inferred archetypes away from the data. Upon orthogonal projection of the data onto a face of the polyhedron, archetypes that are not part of the face increase the projected density close to the middle of the face and thus decrease density in the vicinity of archetypes.

This decreased projected density in the vicinity of archetypes is observed even though density in the high-dimensional polyhedron is uniform. The more archetypes, the smaller the density in the vicinity of archetypes after projecting the data.

Supplementary Note 2 - Archetypes do not represent single cell types

We tested the possibility that the polyhedra described by tumors could be caused by mixing different cell types (cancer cells, fibroblasts, immune cells, ...) in varying proportions rather than evolutionary trade-offs. Two observations suggest that it is unlikely that archetypes correspond to cell types which are mixed in varying proportions in different tumors..

First, mixing cell types in different proportions should produce tumors that describe polyhedra in linear gene expression space [1], but not log gene expression space. We looked for polyhedra in linear gene expression space by exponentiating gene expression right before subtracting the mean gene expression from each gene (see “Fitting polyhedra to tumor gene expression data with ParTI” in Materials and Methods). No significant polyhedra were found in linear gene expression space in any of the 15 cancer types.

Second, if archetypes represent individual cell types mixed in different proportions in different tumors, one archetype should represent pure cancer cells while the other archetypes should represent other cell types present in the tumor (fibroblasts, immune cells, …). Thus, tumor purity should peak at one of the archetypes and monotonically decrease away from this archetype, as cancer cells are increasingly mixed with other cell types. We tested this prediction by analyzing tumor purity, defined as the fraction of cancer cells in a tumor. Purity can be inferred from bulk tumor gene expression profiles by algorithms such as ESTIMATE [2]. We find that purity peaks at several archetypes (cell division, biomass&energy) and is lowest close to the invasion & tissue remodeling archetype (Supplementary Fig. 1C). This observation is not expected if tumors mix cancer cells with other cell types in varying proportions but is consistent with the increased proportion of stromal cells found in tumors close to the invasion & tissue remodeling archetype (Supplementary Data 3).

To determine how the inferred tasks are influenced by clonal heterogeneity, we stratified our analysis according to the Mutant-Allele Tumor Heterogeneity Score (MATH), an established measure of clonal heterogeneity[3,4]. The MATH score is defined as the median absolute deviation of the frequency of mutations found in a tumor. In homogeneous tumors, cancer cells share the same alleles so that the frequency of different mutations tends to be similar and the MATH score is low. The MATH score is high when different mutations occur at different frequencies, as happens in heterogeneous tumors in which multiple lineages of subpopulations of cancer cells coexist.

Analyzing the 25% tumors with highest MATH score in the Metabric cohort, we find the same four archetypes as when analyzing all Metabric tumors together: 1. cell division, 2. tissue remodeling & invasion, 3. biomass & energy, 4. Her2 (Supplementary Fig. 1H). Analyzing Metabric tumors in the lowest MATH score quartile, we find three archetypes (Supplementary Fig. 1H). Two archetypes are shared with heterogeneous tumors (1. Her2, 2.

biomass&energy). The third archetype corresponds to the task of immune interaction.

Thus, ParTI can be applied to both clonally homogeneous and heterogeneous tumors. Most tasks appear to be shared among tumors, while certain tasks are seen when focusing on tumors with specific properties – here homogeneous tumors.

Supplementary Note 3 - Alignment of passenger CNAs to the front and spatial dependencies

In the five cancer types where driver SNVs aligned with the cancer front better than shuffled controls and with the exception of bladder, shuffled controls were as aligned to the cancer front as passenger mutations (Supplementary Fig. 3A). In contrast, passenger CNAs were more aligned with the cancer front than shuffled controls in all 6 cancer types (Supplementary Fig. 3B).

This result is likely explained by the fact that chromosomal amplifications or deletions typically involve a portion of a chromosome that contains many genes. Thus many neighboring genes are amplified or deleted along with the driver gene. One example of this phenomenon is shown on Supplementary Fig. 3C: the driver CNA ATM(-1) (i.e. ATM deletion) is best aligned with the cancer front, and pulls neighboring genes in its wake.

Because of the spatial dependencies of CNAs, the general pattern is that *driver* CNAS are most aligned with the front, followed by CNAs of *other cancer genes*, followed by *passenger* CNAs, followed by *shuffled controls* (Supplementary Fig. 3B).

Supplementary Figures

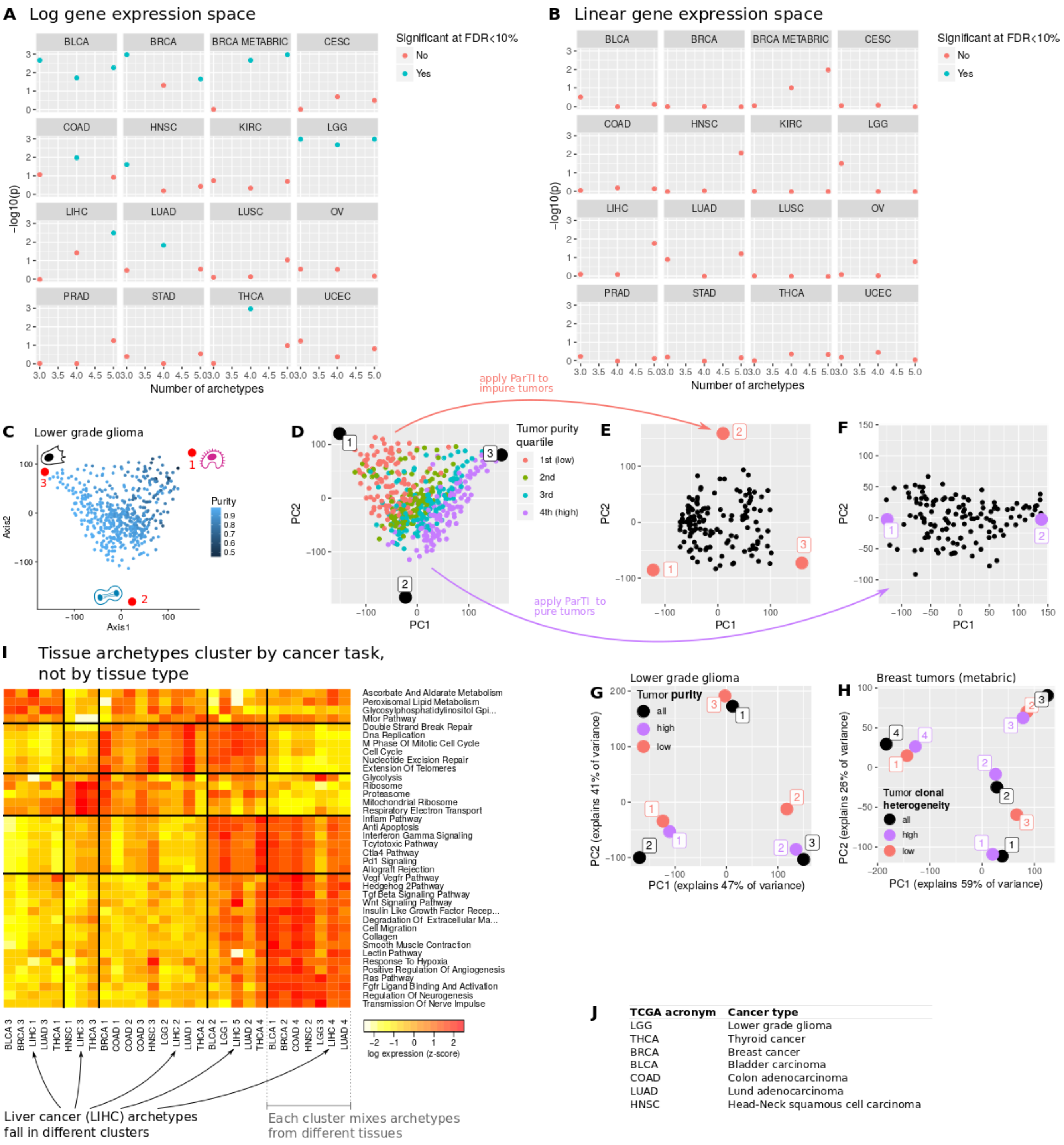

Supplementary Figure 1

A. Statistical significance of polyhedra with 3, 4 and 5 archetypes in primary tumors of 15 cancer types. P-values were computed using the t-ratio test [5,6]. Polyhedra were inferred in log gene expression space. Polyhedra significant at FDR<10% appear in blue. **B.** Same as A., but for polyhedra in linear gene expression space. **C.** Purity score of tumors as computed by ESTIMATE [2] as function of their position relative to the three archetypes of glioma. Each blue dot represents a tumor, red dots represent archetypes. Archetype numbering

corresponds to panel I and Database S1-S3. **D.** To determine how tumor purity affects ParTI's ability to resolve archetypes, we stratify the analysis according to purity of low-grade glioma tumors. **E.** Applying ParTI to the 25% low-grade glioma tumors of lowest purity reveals three archetypes. While a polyhedron is poor fit to the to data, archetypes identified from low purity gliomas match archetypes identified from all glioma (see panel G). **F.** Applying ParTI to the 25% low-grade glioma tumors with highest purity reveals two archetypes. **G.** Principal component analysis comparison of archetypes identified from all gliomas, pure gliomas and impure gliomas shows that the same archetypes are inferred from tumors of different purities. The two archetypes identified from pure tumors match glioma archetypes 2 and 3 whereas all three glioma archetypes are identified from low-purity tumors. These results suggest that ParTI identifies archetypes relevant to a given tumor subset. **H.** Same as D-G, except that metabric breast tumors are stratified by clonal heterogeneity. A principal component projection of archetypes identified from all tumors, clonally heterogeneous tumors and clonally homogeneous tumors shows that tasks can be inferred from clonally homogeneous and heterogeneous tumors. Most tasks are shared among these tumors, while some tasks are only found when focusing on certain tumors.
I. Expression of MSigDB pathways (rows) in archetypes from all cancer types (columns). Archetypes were clustered by Gaussian Mixture Model. Each cluster expresses specific MSigDB pathways. **J.** TCGA code for each cancer type.

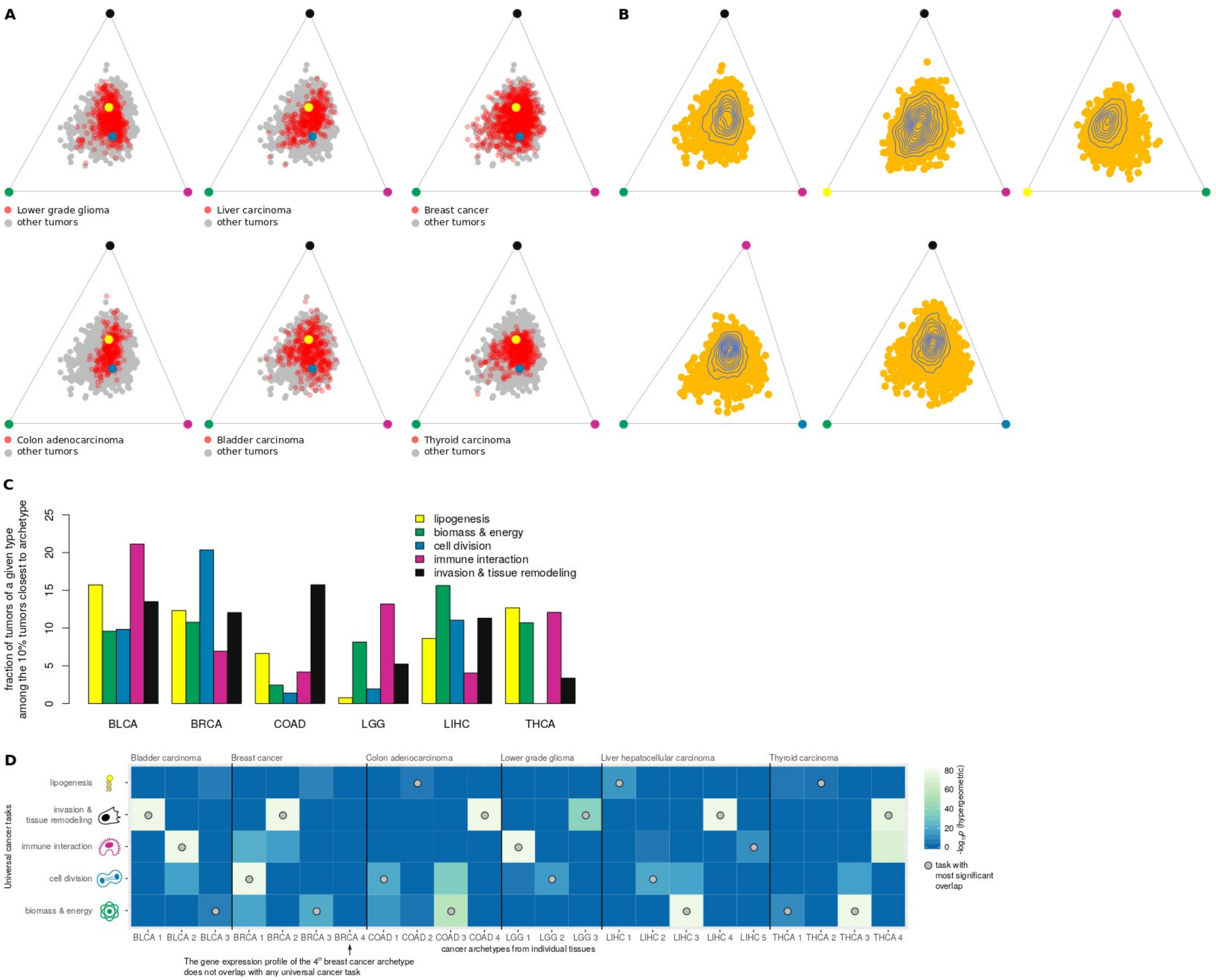
A
Lower grade glioma
other tumors
Liver carcinoma
other tumors
Breast cancer
other tumors
Colon adenocarcinoma
other tumors
Bladder carcinoma
other tumors
Thyroid carcinoma
other tumors
B
C
fraction of tumors of a given type among the 10% tumors closest to archetype
lipogenesis
biomass & energy
cell division
immune interaction
invasion & tissue remodeling
BLCA
BRCA
COAD
LGG
LIHC
THCA
D
Bladder carcinoma
Breast cancer
Colon adenocarcinoma
Lower grade glioma
Liver hepatocellular carcinoma
Thyroid carcinoma
Universal cancer tasks
lipogenesis
invasion & tissue remodeling
immune interaction
cell division
biomass & energy
task with most significant overlap
cancer archetypes from individual tissues
The gene expression profile of the 4th breast cancer archetype does not overlap with any universal cancer task

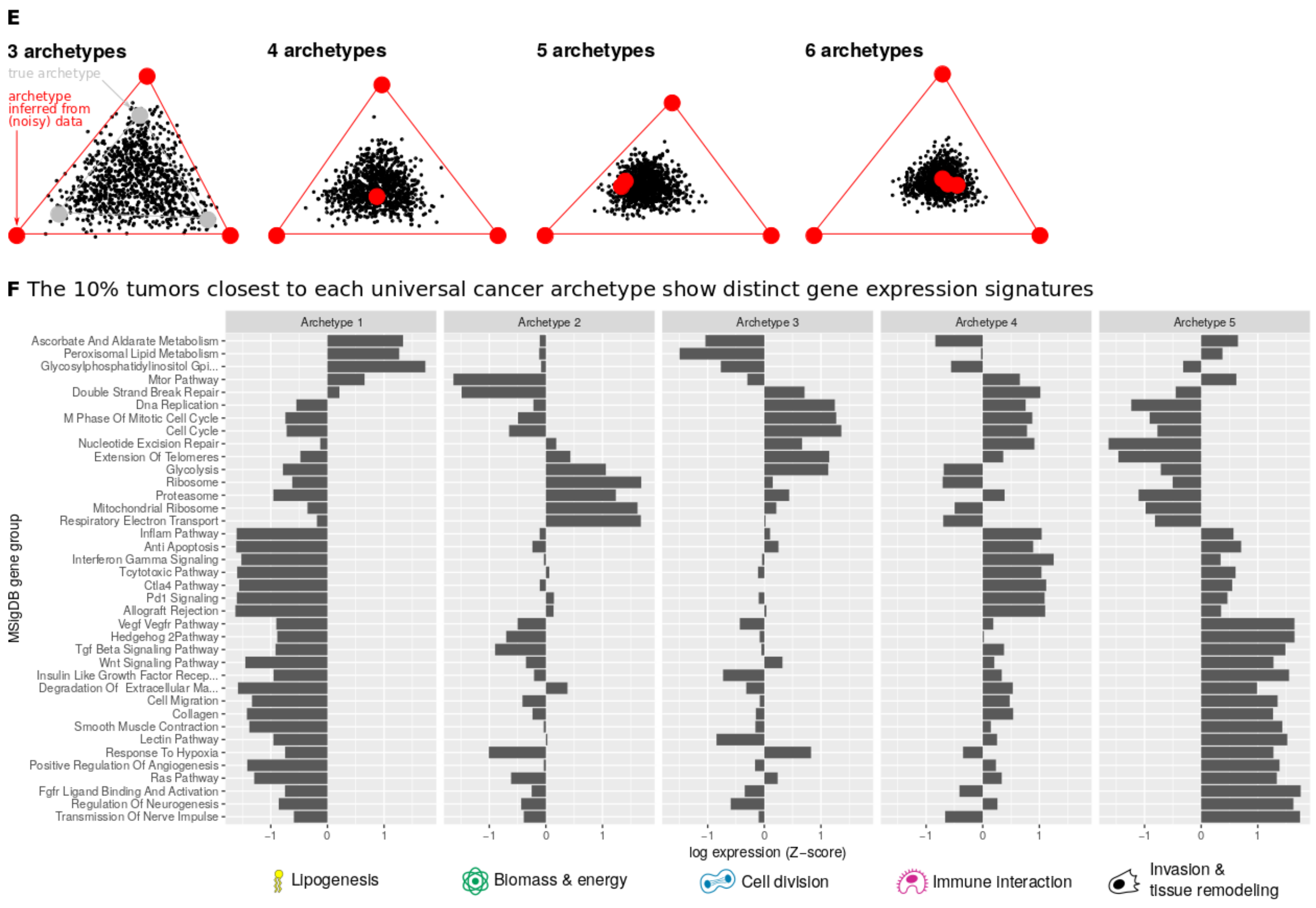

Supplementary Figure 2

A. Tumors from individual tissue types spread out between several universal cancer archetypes. Red dots represent tumors from the tissue-type indicated in each panel, projected on a face of the polyhedron. The polyhedron was fitted to 3180 tumors from 6 tissue types. Grey dots represent other tumors. The remaining colored dots represent the projections of the 5 archetypes on the face. **B.** Density of tumors over the faces of the 5-archetype polyhedron of Fig. 2A. **C.** Tumors of individual tissue types are found close to multiple universal cancer archetypes. Each colored bar represents a cancer task. For example, 10.7% of thyroid tumors (THCA) are found in the 10% tumors from all cancer types closest to biomass&energy archetype. **D.** Tissue-specific archetypes can be assigned to specific universal cancer tasks with statistical significance. We compared the MSigDB pathways upregulated in each tissue-specific archetype (columns) to pathways upregulated in each universal cancer archetype (rows). The statistical similarity between pairs of archetypes was quantified using the hypergeometric test (p-values are color-coded). For each tissue-specific archetype, the most similar and statistically significant universal archetypes is signaled by a gray dot. Except in thyroid, each universal cancer task was only found once in each tissue type. For each tissue-specific archetype, there is a statistically similar universal cancer archetype, except for the 4th archetype of breast cancer (HER2). **E.** Tumors are not found in the immediate vicinity of archetypes is an artifact of visualizing high-dimensional data. Data points were sampled uniformly from polyhedra with

3, 4, 5 and 6 archetypes, Gaussian noise was added, the archetypes determined using ParTI, and the data projected on a face of the polyhedron.. **F.** Expression of MSigDB pathways in tumors close to each universal cancer archetype in gene expression space. Upregulated pathways at each archetype match tissue-specific archetype clusters (Supplementary Fig. 1I) and suggest clear cancer tasks (Table 2).

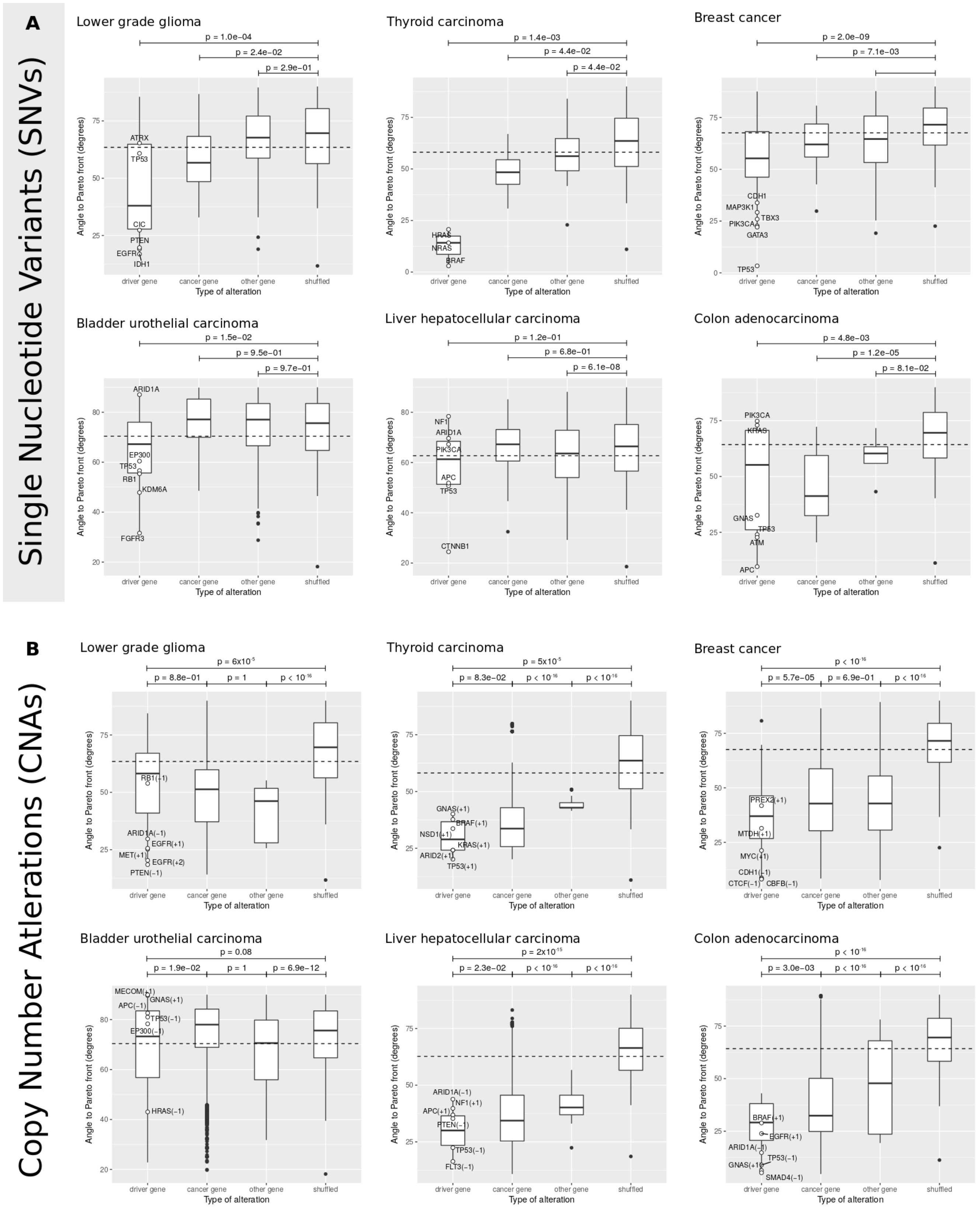

Single Nucleotide Variants (SNVs)
A
Lower grade glioma
p = 1.0e−04
p = 2.4e−02
p = 2.9e−01
ATRX
TP53
CIC
PTEN
EGFR
IDH1
Thyroid carcinoma
p = 1.4e−03
p = 4.4e−02
p = 4.4e−02
HRAS
NRAS
BRAF
Breast cancer
p = 2.0e−09
p = 7.1e−03
CDH1
MAP3K1
TBX3
PIK3CA
GATA3
TP53
Bladder urothelial carcinoma
p = 1.5e−02
p = 9.5e−01
p = 9.7e−01
ARID1A
EP300
TP53
RB1
KDM6A
FGFR3
Liver hepatocellular carcinoma
p = 1.2e−01
p = 6.8e−01
p = 6.1e−08
NF1
ARID1A
PIK3CA
APC
TP53
CTNNB1
Colon adenocarcinoma
p = 4.8e−03
p = 1.2e−05
p = 8.1e−02
PIK3CA
KRAS
GNAS
TP53
ATM
APC
Angle to Pareto front (degrees)
Type of alteration
driver gene
cancer gene
other gene
shuffled
Copy Number Atlerations (CNAs)
B
Lower grade glioma
p = 6x10^-5
p = 8.8e−01
p = 1
p < 10^-16
RB1(+1)
ARID1A(−1)
EGFR(+1)
MET(+1)
EGFR(+2)
PTEN(−1)
Thyroid carcinoma
p = 5x10^-5
p = 8.3e−02
p < 10^-16
p < 10^-16
GNAS(+1)
BRAF(+1)
NSD1(+1)
KRAS(+1)
ARID2(+1)
TP53(+1)
Breast cancer
p < 10^-16
p = 5.7e−05
p = 6.9e−01
p < 10^-16
PREX2(+1)
MTDH(+1)
MYC(+1)
CDH1(−1)
CTCF(−1)
CBFB(−1)
Bladder urothelial carcinoma
p = 0.08
p = 1.9e−02
p = 1
p = 6.9e−12
MECOM(+1)
GNAS(+1)
APC(−1)
TP53(−1)
EP300(−1)
HRAS(−1)
Liver hepatocellular carcinoma
p = 2x10^-15
p = 2.3e−02
p < 10^-16
p < 10^-16
ARID1A(−1)
NF1(+1)
APC(+1)
PTEN(−1)
TP53(−1)
FLT3(−1)
Colon adenocarcinoma
p < 10^-16
p = 3.0e−03
p < 10^-16
p < 10^-16
BRAF(+1)
EGFR(+1)
ARID1A(−1)
TP53(−1)
GNAS(+1)
SMAD4(−1)

C

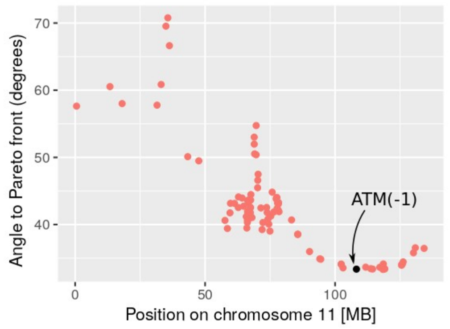

D

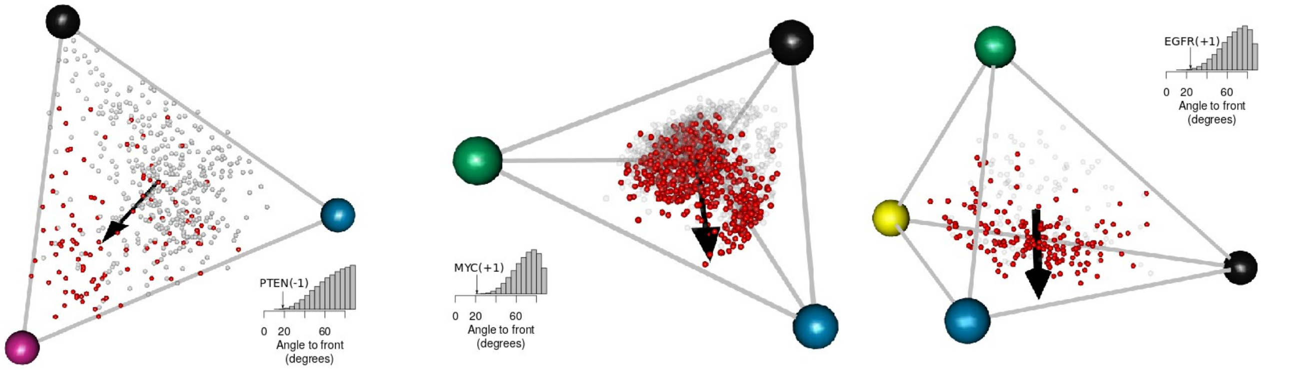

PTEN deletion in lower grade glioma points at the immune interaction archetype

MYC amplification in breast cancer points at the cell division archetype

EGFR amplification in colorectal adenocarcinoma points at the cell division archetype

Supplementary Figure 3

A. Driver SNVs are better aligned to the front of cancer than shuffled controls in glioma, thyroid, breast, bladder and colon. Shown are angle distributions of SNVs in driver genes, cancer genes (genes commonly mutated in cancer but not confidently known as drivers in this tissue), passenger genes and shuffled controls. Differences in distributions were tested using the Mann-Whitney test. **B.** Same as A, but for CNAs. Compared to SNVs, CNAs in cancer genes and other genes aligns better than shuffled controls, a trend that is likely due to chromosomal spatial dependencies (Supplementary Note 3). **C.** There are spatial dependencies in the alignment of CNAs to the Pareto front of cancer. For example, CNA ATM(-1) (i.e. ATM deletion) is best aligned with the cancer front. Other genes located on the same chromosome are less aligned.. **D.** In glioma, *PTEN* deletions push tumors towards the immune interaction archetype. In breast cancer, *MYC* amplification pushes tumors towards to cell division archetype. In colon cancer, *EGFR* amplification pushes tumors towards the cell division archetype.

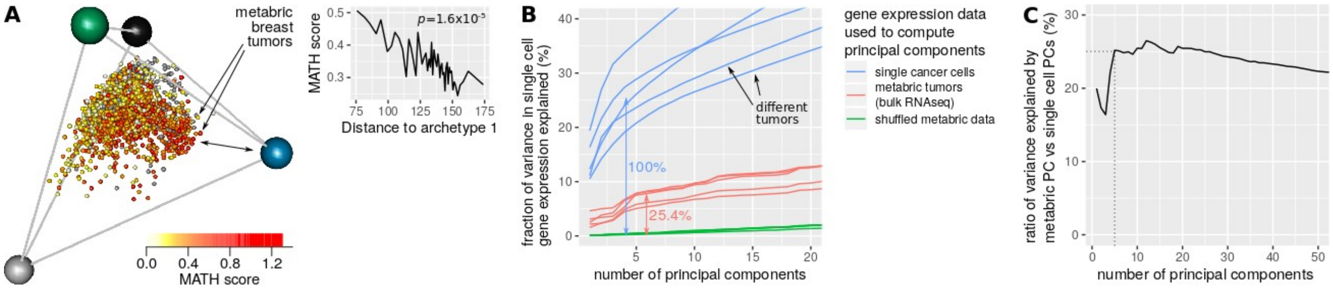

Supplementary Figure 4

A. Variation of clonal heterogeneity over the tetrahedron of metabric breast tumors is inconsistent with tumors being made of different abundances of specialist single cancer cells. If tumor positioning relative to archetypes is set by the abundance of specialist single cells, clonal heterogeneity should be low close to archetypes and high in the center of the tetrahedron. Instead, we find that clonal heterogeneity – quantified by the MATH score [4] – can both be high or low close to archetypes. Clonal heterogeneity is not lowest close to archetypes. Instead, clonal heterogeneity is highest in tumors closest to the cell division archetype. **B.** Inter-tumor diversity explains a significant fraction of intra-tumor heterogeneity in gene expression. The fraction of the variance in single cancer cell gene expression explained by 1-20 principal components (PCs) is plotted for PCs computed on three different data sets: single cell gene expression, metabric tumor gene expression, and shuffled metabric gene expression data. For example, for 5 PCs, metabric PCs explain 25.4% of the variance explained by single cell gene expression PCs. **C.** The ratio of variance explained by metabric PCs over single cell PCs is robust to the number of PCs used. Data: metabric gene expression from Curtis et al., metabric MATH scores from Pereira et al.[3], single cancer cell gene expression from 6 breast tumors from Karaayvaz et al.[7].

Supplementary References

1. Shen-Orr, S. S. *et al.* Cell type-specific gene expression differences in complex tissues. *Nat. Methods* **7**, 287–9 (2010).

2. Yoshihara, K. *et al.* Inferring tumour purity and stromal and immune cell admixture from expression data. *Nat. Commun.* **4**, 2612 (2013).

3. Pereira, B. *et al.* The somatic mutation profiles of 2,433 breast cancers refines their genomic and transcriptomic landscapes. *Nat. Commun.* **7**, 11479 (2016).

4. Mroz, E. A. & Rocco, J. W. MATH, a novel measure of intratumor genetic heterogeneity, is high in poor-outcome classes of head and neck squamous cell carcinoma. *Oral Oncol.* **49**, 211–5 (2013).

5. Shoval, O. *et al.* Evolutionary trade-offs, pareto optimality, and the geometry of phenotype space. *Science (80-.).* **336**, 1157–1160 (2012).

6. Hart, Y. *et al.* Inferring biological tasks using Pareto analysis of high-dimensional data. *Nat. Methods* **12**, 233–235 (2015).

7. Karaayvaz, M. *et al.* Unravelling subclonal heterogeneity and aggressive disease states in TNBC through single-cell RNA-seq. *Nat. Commun.* **9**, (2018).
