# Supplementary material for: Tumor diversity and the trade-off between universal cancer tasks
Source: Nat Commun. 2019 Nov 28;10:5423. doi: 10.1038/s41467-019-13195-1 (PMC6882839; doi:10.1038/s41467-019-13195-1)
Supplement: Supplementary file 3 — Description of Additional Supplementary Files [file 41467_2019_13195_MOESM3_ESM.pdf]

Description of Additional Supplementary Files

File Name: Supplementary Data 1

Description: MSigDB pathways up-regulated in the 5% tumors closest to individual archetypes. Each MSigDB pathway (feature name) is listed with the corresponding archetype. The statistical significance of the up-regulation was quantified using the Mann-Whitney U test. Uncorrected p-values appear in the table, together with median and mean difference in pathway expression of tumors in the first distance bin compared to all other tumors. MSigDB pathways only appear if statistically significant at FDR<10%, and if pathway expression is highest in tumors of the first distance bin. Results from the combined analysis of 3180 tumors from 6 cancer types appear on the sheet labeled 'All 6 cancer types'. Also shown are individual genes up-regulated in tumors closest to individual archetypes. MSigDB pathways up-regulated when analyzing tumors from individual tissue-types separately appear in the different sheets of the spreadsheet.

File Name: Supplementary Data 2

Description: Qualitative clinical features over-represented in the 5% tumors closest to individual archetypes. Each clinical feature is listed with the corresponding archetype. The statistical significance of the over-representation was quantified using the hypergeometric test. Uncorrected p-values appear in the table. Clinical features only appear if statistically significant at FDR<10%, and if the feature is most frequent among tumors of the first distance bin. Results from the combined analysis of 3810 tumors from 6 cancer types appear on the sheet labeled 'All 6 cancer types'. Clinical features overrepresented when analyzing tumors from individual tissue-types separately appear in the different sheets of the spreadsheet. The definition of the clinical feature can be found at https://docs.gdc.cancer.gov/Data_Dictionary/viewer/.

File Name: Supplementary Data 3

Description: Quantitative clinical features which take high values in the 5% tumors closest to individual archetypes. Each clinical feature is listed with the corresponding archetype. The statistical significance of the difference between tumors in the first distance bin compared to other tumors was quantified using the Mann-Whitney U test. Uncorrected p-values appear in the table. Clinical features only appear if statistically significant at FDR<10%, and if the feature has highest value in tumors of the first distance bin. Results from the combined analysis of 3180 tumors from 6 cancer types appear on the sheet labeled 'All 6 cancer types'. Clinical features overrepresented when analyzing tumors from individual tissue-types separately appear in the different sheets of the spreadsheet. The definition of the clinical feature can be found at
https://docs.gdc.cancer.gov/Data_Dictionary/viewer/.

File Name: Supplementary Data 4

Description: Driver SNVs significantly aligned with the Pareto front in different cancer types (FDR<10%). For each cancer type, driver genes are listed together with the archetype / edge / face the driver points to. Also shown is the number of tumors in which the SNV was found.

File Name: Supplementary Data 5

Description: Driver CNAs significantly aligned with the Pareto front in different cancer types (FDR<10%). For each cancer type, driver CNAs are listed together with the archetype / edge / face the driver points to. A CNA can be a strong deletion (-2), a weak deletion (-1), a weak amplification (+1) or a strong amplification (+2).

File Name: Supplementary Data 6

Description: For a given task, gene expressed by archetypes from different tissues have a tissue-specific flavor. For each task and each tissue, we list gene groups
(MSigDB) upregulated at the tissue archetype but not at the universal archetype.
