# Supplementary material for: Tumor diversity and the trade-off between universal cancer tasks
Source: Nat Commun. 2019 Nov 28;10:5423. doi: 10.1038/s41467-019-13195-1 (PMC6882839; doi:10.1038/s41467-019-13195-1)
Supplement: Supplementary file 10 — Reporting Summary [file 41467_2019_13195_MOESM10_ESM.pdf]

nature research

Corresponding author(s): Uri Alon; Carlos Caldas

Last updated by author(s): Sep 23, 2019

Reporting Summary

Nature Research wishes to improve the reproducibility of the work that we publish. This form provides structure for consistency and transparency in reporting. For further information on Nature Research policies, see Authors & Referees and the Editorial Policy Checklist.

Statistics

For all statistical analyses, confirm that the following items are present in the figure legend, table legend, main text, or Methods section.

n/a	Confirmed	
☐	☒	The exact sample size (*n*) for each experimental group/condition, given as a discrete number and unit of measurement
☒	☐	A statement on whether measurements were taken from distinct samples or whether the same sample was measured repeatedly
☐	☒	The statistical test(s) used AND whether they are one- or two-sided<br>*Only common tests should be described solely by name; describe more complex techniques in the Methods section.*
☒	☐	A description of all covariates tested
☐	☒	A description of any assumptions or corrections, such as tests of normality and adjustment for multiple comparisons
☐	☒	A full description of the statistical parameters including central tendency (e.g. means) or other basic estimates (e.g. regression coefficient) AND variation (e.g. standard deviation) or associated estimates of uncertainty (e.g. confidence intervals)
☒	☐	For null hypothesis testing, the test statistic (e.g. *F*, *t*, *r*) with confidence intervals, effect sizes, degrees of freedom and *P* value noted<br>*Give P values as exact values whenever suitable.*
☒	☐	For Bayesian analysis, information on the choice of priors and Markov chain Monte Carlo settings
☒	☐	For hierarchical and complex designs, identification of the appropriate level for tests and full reporting of outcomes
☒	☐	Estimates of effect sizes (e.g. Cohen's *d*, Pearson's *r*), indicating how they were calculated

Our web collection on statistics for biologists contains articles on many of the points above.

Software and code

Policy information about availability of computer code

Data collection	This article did not collect new data.
Data analysis	The code to reproduce the analyses can be downloaded at http://dx.doi.org/10.17632/2r9h9xzwm3.1. Analyses were performed in R 3.4 and Matlab R2016b.

For manuscripts utilizing custom algorithms or software that are central to the research but not yet described in published literature, software must be made available to editors/reviewers. We strongly encourage code deposition in a community repository (e.g. GitHub). See the Nature Research guidelines for submitting code & software for further information.

Data

Policy information about availability of data

All manuscripts must include a data availability statement. This statement should provide the following information, where applicable:

- Accession codes, unique identifiers, or web links for publicly available datasets
- A list of figures that have associated raw data
- A description of any restrictions on data availability

Source data files to reproduce the analyses can be downloaded at:
http://dx.doi.org/10.17632/2r9h9xzwm3.1
The TCGA data used in this study was downloaded from RSEM-normalized HiSeqV2 gene expression data (log2 RPKMs) from the TCGA data portal. The TCGA data portal is now retired but the data can be retrieved from the Genomics Data Commons portal of the National Cancer Institute [https://portal.gdc.cancer.gov/]. The starting point of our analyses were the 'genomicMatrix' files which contain expression levels for 20530 human genes in 15 cancer types. We considered all cancer types with at least 250 primary tumor samples: BLCA [https://portal.gdc.cancer.gov/projects/TCGA-BLCA], BRCA [https://portal.gdc.cancer.gov/projects/TCGA-BRCA], CESC [https://portal.gdc.cancer.gov/projects/TCGA-CESC], COAD [https://portal.gdc.cancer.gov/projects/TCGA-COAD], HNSC [https://portal.gdc.cancer.gov/projects/TCGA-HNSC], KIRC [https://portal.gdc.cancer.gov/projects/TCGA-KIRC], LGG [https://portal.gdc.cancer.gov/projects/TCGA-LGG], LIHC [https://portal.gdc.cancer.gov/projects/TCGA-LIHC], LUAD [https://portal.gdc.cancer.gov/projects/TCGA-LUAD], LUSC [https://portal.gdc.cancer.gov/projects/TCGA-LUSC],

OV [https://portal.gdc.cancer.gov/projects/TCGA-OV], PRAD [https://portal.gdc.cancer.gov/projects/TCGA-PRAD], STAD [https://portal.gdc.cancer.gov/projects/TCGA-STAD], THCA [https://portal.gdc.cancer.gov/projects/TCGA-THCA], UCEC [https://portal.gdc.cancer.gov/projects/TCGA-UCEC]. The data for the 1970 tumors of the METABRIC data was downloaded from the cBio portal [http://www.cbioportal.org/study/summary?id=brca_metabric]. We describe in detail how we obtained and processed the data in the Methods.

Field-specific reporting

Please select the one below that is the best fit for your research. If you are not sure, read the appropriate sections before making your selection.

☒ Life sciences ☐ Behavioural & social sciences ☐ Ecological, evolutionary & environmental sciences

For a reference copy of the document with all sections, see nature.com/documents/nr-reporting-summary-flat.pdf

Life sciences study design

All studies must disclose on these points even when the disclosure is negative.

Sample size	No sample size calculation were performed as the present studied re-analyzed publicly available genomics datasets.
Data exclusions	Only primary tumors were used in our analyses. We excluded healthy tissues, as well as local and distant metastases because these samples were less numerous than primary tumors (the focus of the TCGA and METABRIC studies), and because selective pressure and tissue biology may differ for these, thus complicating the interpretation of the data.
Replication	Experimental findings were not reproduced: the present study is a purely computational study.
Randomization	No data were collected, hence no randomization could be applied.
Blinding	No blinding was possible because the aim of the study was to determine cancer archetypes and correlate these to known tumor properties. To do so, the data could be blinded during the analysis.

Reporting for specific materials, systems and methods

We require information from authors about some types of materials, experimental systems and methods used in many studies. Here, indicate whether each material, system or method listed is relevant to your study. If you are not sure if a list item applies to your research, read the appropriate section before selecting a response.

Materials & experimental systems

n/a	Involved in the study	
☒	☐	Antibodies
☒	☐	Eukaryotic cell lines
☒	☐	Palaeontology
☒	☐	Animals and other organisms
☒	☐	Human research participants
☒	☐	Clinical data

Methods

n/a	Involved in the study	
☒	☐	ChIP-seq
☒	☐	Flow cytometry
☒	☐	MRI-based neuroimaging
